# Effects of Caffeic Acid Supplementation on Human Sperm Against In Vitro-Induced Oxidative Stress: Nrf2 Molecular Pathway

**DOI:** 10.3390/antiox15010133

**Published:** 2026-01-20

**Authors:** Laura Liguori, Cinzia Signorini, Giulia Collodel, Caterina Marcucci, Elena Moretti

**Affiliations:** Department of Molecular and Developmental Medicine, University of Siena, 53100 Siena, Italy; laura.liguori@student.unisi.it (L.L.); cinzia.signorini@unisi.it (C.S.); caterin.marcucci@student.unisi.it (C.M.); elena.moretti@unisi.it (E.M.)

**Keywords:** acrosome, caffeic acid, DNA integrity, F_2_-isoprostanes, human semen, Nrf2 expression, oxidative stress, sperm motility

## Abstract

Oxidative stress (OS) is a major cause of defective sperm function. During laboratory handling, gametes are exposed to OS, potentially mitigated by in vitro antioxidant supplementation. This study evaluates the protective role of caffeic acid (CAF) on basal human semen and under induced OS. First, six semen samples from normozoospermic donors were incubated with CAF concentrations ranging from 50 to 500 µM at 37 °C for 2 h. Sperm motility and DNA integrity (acridine orange) were evaluated. Then, ten semen samples were divided into four aliquots and incubated, respectively, with CAF at 100 µM, H_2_O_2_ at 2 mM, or H_2_O_2_ at 2 mM + CAF at 100 µM, or untreated. Motility, DNA integrity, acrosome status (*Pisum sativum* agglutinin), OS quantified by F_2_-isoprostanes (ELISA), and expression of Nrf2, Keap1, and HO-1 (qRT-PCR) were assessed. CAF at 100 µM improved progressive motility without damaging DNA and was selected for subsequent experiments. CAF showed protective effects on sperm damage induced by H_2_O_2_ treatment, restoring motility, DNA integrity, and acrosome status and reducing F_2_-isoprostane levels. Nrf2 and HO-1 expression were upregulated by CAF, downregulated by H_2_O_2_, and restored by the co-treatment. CAF supplementation may protect human spermatozoa during in vitro handling by reducing OS, improving several sperm parameters, with a possible mechanism of action involving the Nrf2 pathway.

## 1. Introduction

In recent years, increasing attention has been devoted to the evidence that oxidative stress (OS) plays a pivotal role in the etiology of male infertility [1]. OS refers to a condition arising from an imbalance between the levels of reactive oxygen species and the antioxidant defenses present in the organism [2,3]. As a result, substantial research has focused on identifying natural compounds with antioxidant properties that could be used to treat diseases primarily driven by OS [3,4].

The considered compound of this study is caffeic acid (CAF), a naturally occurring polyphenol found in various plant-based sources [5].

The chemical structure of CAF is thought to play a crucial role in its ability to scavenge free radicals, as it allows for the delocalization of unpaired electrons. The antioxidant activity, however, represents only one of its many beneficial biological effects. Indeed, CAF has shown antibacterial activity [6,7], antiproliferative effects against several cancer cells by acting on different receptors and signaling pathways [8,9,10,11,12], and anti-diabetic and anti-neuroinflammatory properties [13,14].

With regard to male reproductive health, most available studies on CAF have been conducted in animal models and demonstrated that CAF treatment was beneficial for sperm quality both in vivo [15] and in vitro during freezing–thawing procedures [16,17].

This study focused on investigating the in vitro effects of CAF on human semen, addressing a notable gap in the current scientific literature. Human spermatozoa represent an excellent in vitro model because they are easy to collect and allow straightforward assessment of parameters such as motility [18]. Spermatozoa possess minimal intracellular antioxidant defenses and exhibit limited transcriptional activity. Most antioxidant capacity resides in the seminal plasma, which modulates and mitigates the effects of in vitro-induced OS. This feature enables the development of controlled OS models using defined concentrations of oxidant stressors, thereby facilitating a more effective evaluation of antioxidant compounds and their biological effects. These characteristics make spermatozoa a particularly sensitive and suitable model for testing the effects of bioactive compounds such as CAF. Many routine procedures performed in reproductive laboratories cause OS in sperm cells. These include exposure to visible light, fluctuations in oxygen tension and temperature, pH variations, and cryopreservation [19,20]. The cell damage due to OS can be assessed by the quantification of biomarkers such as F_2_-isoprostanes (F_2_-IsoPs), identified as reliable and specific indicators of lipid peroxidation in biological tissues [21,22].

CAF has been demonstrated to influence the expression of various genes in different cell models [5,12], including nuclear factor-E2-related factor 2 [23] (NFE2L2, hereafter referred to by its transcription factor name, Nrf2), widely recognized as a key regulator of endogenous antioxidant responses [24]. Under physiological conditions, Keap1 directs Nrf2 for ubiquitin-dependent degradation, thereby repressing Nrf2-dependent gene expression [25,26]. In response to OS, the newly synthesized Nrf2 enters the nucleus, where it forms a heterodimer with small Maf proteins (musculoaponeurotic fibrosarcoma oncogene homologs). The resulting Nrf2-Maf complex selectively binds to antioxidant response elements (AREs) in the promoter regions of target genes such as heme oxygenase-1 (HO-1)—hereafter referred to by its protein name, HO-1—thereby activating Nrf2-dependent transcription [26]. Recently, Signorini and colleagues [27] reported an upregulation of Nrf2 expression following in vitro treatment of human spermatozoa with chlorogenic acid, a compound derived from the esterification of CAF and quinic acid, further supporting the involvement of the Nrf2 pathway in mediating the antioxidant effects of phenolic compounds.

This study aimed to evaluate the effects of CAF on human semen and to assess its potential biological activity. First, different concentrations of CAF were tested on semen samples from normozoospermic donors, evaluating sperm motility and DNA integrity, in order to identify the optimal concentration for subsequent experiments. Then, the effects of CAF were assessed in human semen samples exposed in vitro to OS induced by H_2_O_2_. The endpoints assessed included sperm motility, DNA integrity, acrosome status, levels of F_2_-IsoPs, and the gene expression of the Keap1-Nrf2 pathway.

## 2. Materials and Methods

### 2.1. Study Design

The study was conducted in two distinct steps:

- Step 1: evaluation of the dose-dependent effects of CAF on human normal semen samples;
- Step 2: investigation of the capacity of CAF to counteract the H_2_O_2_-induced OS in vitro in human normal semen samples.

### 2.2. Donors and Semen Sample Analysis

Semen samples were obtained from 16 donors aged 19–35 years who attended the Department of Molecular and Developmental Medicine at the University of Siena (Italy). The study protocol was approved by the Ethics Committee of the Siena University Hospital (ID CEAVSE 25612). All participants provided written informed consent prior to enrolment in this study. Specimens were collected following a period of 3 to 5 days of ejaculatory abstinence. In accordance with the World Health Organization (WHO) guidelines [28], semen parameters including sperm concentration, motility, vitality, pH, and morphology were evaluated.

### 2.3. Step 1: Evaluation of the Dose-Dependent Effects of CAF on Human Normal Semen Samples

Different concentrations of CAF were tested in 6 semen samples. Semen samples were divided into aliquots supplemented with 50 μM, 100 μM, 150 μM, 200 μM, and 500 μM CAF (Sigma-Aldrich, St. Louis, MO, USA) diluted in distilled water and incubated for two hours at 37 °C. An aliquot without compound supplementation served as an internal control. Following the incubation period, sperm motility was quantified in strict adherence to the WHO laboratory manual criteria [28]. DNA integrity was evaluated with the acridine orange (AO) assay.

#### AO Assay to Analyze the DNA Integrity

The AO test evaluates the susceptibility of sperm nuclear DNA to acid-induced denaturation in situ by quantifying the metachromatic shift of AO fluorescence from green, indicative of double-stranded (ds) DNA, to red, indicative of denatured DNA. Smeared sperm slides were processed according to Tejada and colleagues [29] and immediately examined with a Leitz Aristoplan fluorescence Microscope (Leica, Wetzlar, Germany) equipped with a 490 nm excitation source and 530 nm barrier filter, at 1000× magnification. A minimum of 300 sperm were evaluated, and the results are reported as the percentage of sperm with dsDNA.

### 2.4. Step 2: Investigation of the Capacity of CAF to Counteract the H_2_O_2_-Induced OS In Vitro in Normal Human Semen Samples

Based on the results of the dose–response analysis (step 1), semen samples from 10 donors were divided into four aliquots and treated as follows:

- CTR, control aliquot with no supplementation;
- CAF, aliquot supplemented with CAF at 100 μM;
- H_2_O_2_, aliquot supplemented with H_2_O_2_ at 2 mM to induce OS;
- H_2_O_2_ + CAF, aliquot supplemented with both CAF at 100 μM and H_2_O_2_ at 2 mM.

Aliquots were incubated at 37 °C for 2 h. At the end of the incubation, motility was evaluated according to the WHO guidelines [28]. A portion of each aliquot was mixed with an equal volume of phosphate-buffered saline (PBS) and centrifuged at 400× *g* for 10 min. The resulting pellets were resuspended in PBS, smeared onto glass slides, and fixed using two different protocols: in the first one, Carnoy’s solution was used for the evaluation of DNA integrity by the AO test, and in the second one, cold methanol (20 min at −20 °C) followed by cold acetone (5 min at −20 °C) was used for acrosome assessment. The remaining fraction of each aliquot was centrifuged at 400× *g* for 10 min to separate seminal plasma from the spermatozoa; then, both fractions were stored at −80 °C for further analyses. Seminal plasma was used for the quantification of F_2_-IsoPs using an ELISA kit, while the cell pellets were processed for the gene expression analysis of Nrf2, Keap1, and HO-1.

#### 2.4.1. Evaluation of Acrosome with *Pisum sativum* Agglutinin

TRITC-conjugated *Pisum sativum* agglutinin (PSA, Vector Laboratories Inc., Burlingame, CA, USA) is a lectin that recognizes the carbohydrates of glycoproteins and is used to differentiate acrosome-intact from acrosome-reacted (or acrosome-damaged) human spermatozoa. The smeared slides fixed in methanol and acetone, as previously described, were rinsed in PBS for 10 min, incubated for 30 min (in the dark at room temperature) with TRITC-PSA solution diluted 1:1000 in PBS, and rinsed again in PBS for 15 min. The nuclei of the spermatozoa were stained with 4,6-diamidino-2-phenylindole (DAPI, 1:20,000 in methanol; Sigma-Aldrich, St. Louis, MO, USA), for 2 min, in the dark at room temperature. The slides were mounted with DABCO and observed with a Leica DMI 6000 fluorescence microscope (Leica Microsystems, Wetzlar, Germany). A Leica AF6500 Integrated System for Imaging and Analysis (Leica Microsystem, Wetzlar, Germany) enables image acquisition. Spermatozoa displaying a uniformly stained red acrosomal cap were classified as acrosome-intact. Those exhibiting a shrunken, shortened, or misshapen acrosome were categorized as acrosome-altered spermatozoa, while spermatozoa lacking any visible acrosomal staining were classified as acrosome-absent. A minimum of 300 spermatozoa were evaluated per sample, and the results are reported as percentages.

#### 2.4.2. F_2_-IsoP Quantification

F_2_-isoprostanes were quantified by measuring 8-iso prostaglandin F2α (8-iso PGF2α; hereafter referred to as 8-isoprostane) in seminal plasma using a commercially available ELISA kit (8-Isoprostane ELISA Kit, Cayman Chemical, Ann Arbor, MI, USA). The assay is based on a competitive enzyme immunoassay employing a specific anti-8-isoprostane antibody, an 8-isoprostane–AChE tracer, and Ellman’s reagent. Ninety-six-well plates were pre-coated with the capture antibody (mouse anti-rabbit IgG). Absorbance was measured spectrophotometrically at 405 nm, and sample concentrations were calculated from a standard curve generated using 8-isoprostane standards ranging from 0.8 to 500 pg/mL. Each sample was analyzed in triplicate, and results are expressed as pg/mL.

#### 2.4.3. Gene Expression Analysis

Total RNA was isolated from ejaculated spermatozoa using the PureLink^®^ RNA Mini Kit (Thermo Fisher Scientific, Waltham, MA, USA) according to the manufacturer’s protocol. RNA purity and concentration were assessed by measuring A260/A280 absorbance ratios with a Thermo Scientific™ NanoDrop™ One/OneC Microvolume UV–Vis spectrophotometer (Thermo Fisher Scientific, Waltham, MA, USA). Complementary DNA (cDNA) was synthesized from 500 ng of total RNA per sample using the High-Capacity cDNA Reverse Transcription Kit (Applied Biosystems, Waltham, MA, USA).

Quantitative reverse transcription polymerase chain reaction (qRT-PCR) was then performed to evaluate the mRNA expression levels of Nrf2, Keap1, and HO-1 in sperm cells. Gene expression analysis was carried out using specific PrimePCR™ SYBR™ Green assays (Keap1 qHsaCID0017511; NFE2L2 qHsaCED0038543; HO-1 qHsaCID0022141; Bio-Rad Laboratories, Inc., Hercules, CA, USA) on a QuantStudio™ 5 Real-Time PCR System (Thermo Fisher Scientific, Waltham, MA, USA). To normalize target gene expression, mRNA levels of the housekeeping gene glyceraldehyde-3-phosphate dehydrogenase (GAPDH; PrimePCR™ SYBR™ Green Assay GAPDH qHsaCED0038674, Bio-Rad Laboratories, Inc., Hercules, CA, USA) were simultaneously measured in all samples.

Non-template controls and reverse-transcription negative samples were included and run in triplicate as negative controls to ensure the absence of contamination and to verify the specificity of the amplification reaction. The thermal cycling protocol was performed as follows: initial activation of uracil-DNA glycosylase (UDG) at 50 °C for 2 min, followed by enzyme activation at 95 °C for 2 min and by 40 amplification cycles, each consisting of denaturation at 95 °C for 15 s and annealing at 60 °C for 1 min. Fluorescent quantitative analysis was performed by calculating the difference in cycle threshold (∆Ct) between the target genes and the reference gene GAPDH. Relative changes in Keap1, Nrf2, and HO-1 mRNA expression were determined using the 2^−∆∆Ct^ method. All qRT-PCRs were conducted in triplicate.

#### 2.4.4. Statistical Analysis

Distribution normality was examined using the Kolmogorov–Smirnov test. Comparisons between two groups (step 1) were performed with the Mann–Whitney U test. For analyses involving more than two groups (step 2), differences among groups were initially evaluated using the Kruskal–Wallis test, followed by Dunnett’s post hoc test for multiple pairwise comparisons. Results are expressed as median (25th–75th percentile, interquartile range: IQR). A *p* value below 0.05 was considered statistically significant. All statistical analyses were conducted using GraphPad Prism version 8.4.2.

## 3. Results

### 3.1. Step 1: Evaluation of the Dose-Dependent Effects of CAF on Human Normal Semen Samples

**Figure 1:**
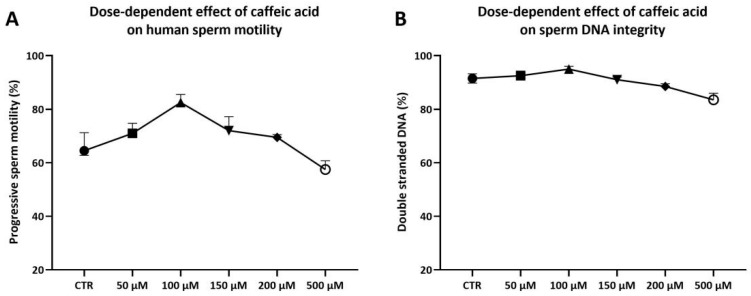
Medians (IQRs) of percentages of progressive sperm motility (**A**) and DNA integrity (**B**) of human sperm after treatments for two hours at 37 °C with increasing concentrations of caffeic acid (range 0–500 μM). CTR indicates untreated aliquot.

The six semen samples used to study the CAF dose-dependent effects showed sperm parameters above the 25th centile [28]. Each sample was divided into six aliquots: five aliquots were treated with increasing concentrations of CAF (50 μM, 100 μM, 150 μM, 200 μM, and 500 μM), while the sixth aliquot was incubated under the same conditions without treatment and served as a control (CTR). Following incubation, progressive sperm motility and DNA integrity were assessed. Treatment with CAF at 100 μM resulted in an increase in progressive motility (82.50% [80.75–85.50%], Figure 1A), calculated as the sum of the percentages of rapid and slow progressive sperm motility. In contrast, sperm treated with CAF at 50 μM (71.00% [67.25–74.75%]), 150 μM (72.00% [67.25–77.25%]), or 200 μM (69.50% [67.25–70.50%], Figure 1A) exhibited motility values similar to CTR (64.50% [62.75–71.25%], Figure 1A); a reduction in motility was observed in spermatozoa exposed to CAF at 500 μM (57.50% [54.75–60.75%], Figure 1A).

None of the tested CAF concentrations exhibited any detrimental effects on DNA integrity (CTR: 91.50% [89.75–93.25%]; 50 μM: 92.50% [89.50–94.25%]; 100 μM: 95.00% [93.25–96.00%]; 150 μM: 91.00% [89.50–92.00%]; 200 μM: 88.50% [87.75–89.50%]; 500 μM: 83.50% [82.75–86.00%], Figure 1B).

### 3.2. Step 2: Investigation of the Capacity of CAF to Counteract the H_2_O_2_-Induced OS In Vitro in Normal Human Semen Samples

**Figure 2:**
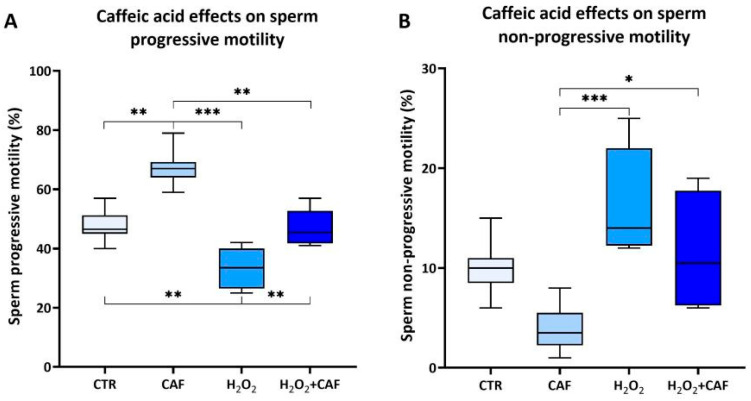
Medians (IQRs) of percentages of progressive sperm motility (**A**) and non-progressive sperm motility (**B**) after treatment for two hours at 37 °C with 100 μM caffeic acid (CAF), 2 mM H_2_O_2_, and combined treatment (H_2_O_2_ + CAF). CTR: untreated aliquot. *: *p* < 0.05; **: *p* < 0.01; ***: *p* < 0.001.

In the second step of the study, ten semen samples with sperm parameters above the 25th percentile [28] were divided into four aliquots, as detailed in the Section 2: CTR, CAF, H_2_O_2_, and H_2_O_2_ + CAF. Sperm motility was assessed [28] as the primary functional parameter. Exposure to CAF resulted in a statistically significant increase in the proportion of progressively motile spermatozoa (67.00% [64.00–69.25%]) compared to the CTR (46.50% [45.00–51.25%], *p* < 0.01), H_2_O_2_ (33.50% [26.50–40.00%], *p* < 0.001), and H_2_O_2_ + CAF (45.50% [41.75–52.75%], *p* < 0.01) aliquots (Figure 2A). A significant reduction in non-progressive sperm motility was observed in the CAF aliquot (3.50% [2.25–5.50%]) with respect to that of both the H_2_O_2_ (14.00% [12.25–22.00%], *p* < 0.001) and H_2_O_2_ + CAF (10.50% [6.25–17.75%], *p* < 0.05) aliquots (Figure 2B). No statistically significant differences in non-progressive sperm motility were detected between the CAF and CTR (10.00% [8.5–11.00%]) groups (Figure 2B).

**Figure 3:**
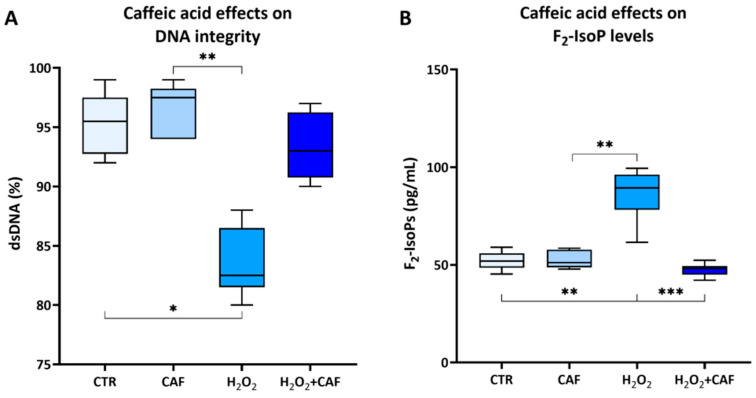
Medians (IQRs) of the percentages of double-stranded (ds) DNA (**A**) and seminal F_2_-isoprostane (F_2_-IsoP, pg/mL) levels (**B**) after treatment for two hours at 37 °C with 100 μM caffeic acid (CAF), 2 mM H_2_O_2_, and combined treatment (H_2_O_2_ + CAF). CTR indicates untreated aliquot. DNA integrity was evaluated using an acridine orange test, scoring a minimum of 300 spermatozoa; the F_2_-IsoP levels were assessed by ELISA. *: *p* < 0.05; **: *p* < 0.01; ***: *p* < 0.001.

The in vitro exposure to H_2_O_2_ to induce OS led to a significant decrease in the percentage of spermatozoa with dsDNA (82.50% [81.50–86.50%]) compared to both the untreated spermatozoa (95.50% [92.75–97.50%], *p* < 0.05), and the spermatozoa treated with CAF at 100 µM (97.50% [94.00–98.25%), *p* < 0.01, Figure 3A). Concurrently, H_2_O_2_-treated samples exhibited a significant increase in F_2_-IsoP levels (89.38% [78.22–96.15%]), a well-established biomarker of lipid peroxidation (free radical-induced oxidative degradation of lipids), compared to all the other samples (CTR: 51.92% [48.46–55.94%], *p* < 0.01; CAF: (51.14% [48.75–57.78%], *p* < 0.01; H_2_O_2_ + CAF: 48.07% [45.03–49.39%], *p* < 0.001), which were similar to each other (Figure 3B).

**Figure 4:**
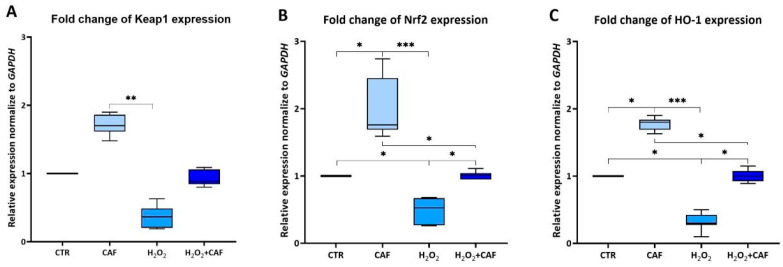
Medians (IQRs) of relative Keap1 (**A**), Nrf2 (**B**), and HO-1 (**C**) expression normalized to GAPDH after treatment for two hours at 37 °C with 100 μM caffeic acid (CAF), 2 mM H_2_O_2_, and combined treatment (H_2_O_2_ + CAF). CTR indicates untreated aliquot. *: *p* < 0.05; **: *p* < 0.01; ***: *p* < 0.001.

The expression levels of Keap1, Nrf2, and HO-1 were quantified by qRT-PCR. The expression patterns of the analyzed genes showed a similar trend across all the considered samples (Figure 4A–C). The treatment with CAF led to a significant increase in Nrf2 and HO-1 expression in spermatozoa compared to the CTR (*p* < 0.05; Figure 4B,C); keap-1 expression also increased, although nonsignificantly. These observations suggest a potential role of CAF in the activation of the Nrf2 pathway. The treatment of spermatozoa with H_2_O_2_ caused in a downregulation in the expression of all three analyzed genes (Figure 4A–C). While the H_2_O_2_ + CAF combination resulted in a significant recovery of sperm Nrf2 and HO-1 expression with respect to H_2_O_2_-treated samples (*p* < 0.05), Keap1 expression exhibited a similar trend, although the change did not reach statistical significance. In any case, the expression of all three considered genes in the samples treated with H_2_O_2_ + CAF reached similar values to CTR.

**Figure 5:**
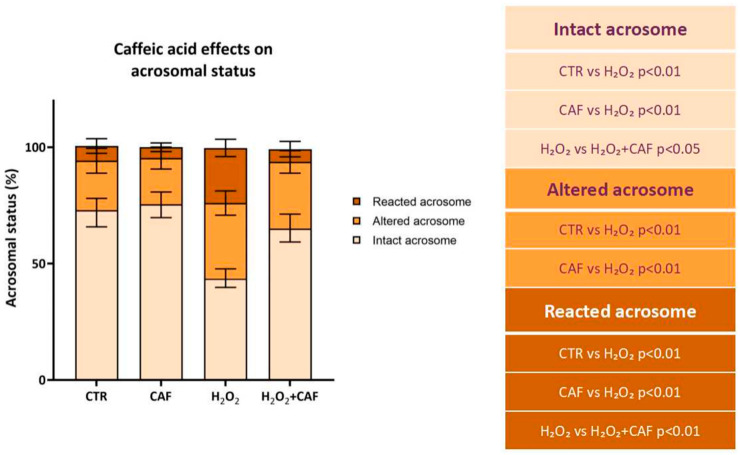
Median (IQR) of percentages of intact, altered, and reacted acrosomes after treatment for two hours at 37 °C with 100 μM caffeic acid (CAF), 2 mM H_2_O_2_, and combined treatment (H_2_O_2_ + CAF). CTR indicates untreated aliquot. The acrosomes were evaluated with *Pisum sativum* agglutinin conjugated with TRITC, scoring a minimum of 300 spermatozoa per sample. The table reports the significant comparisons between pairs.

Finally, the acrosome was assessed through PSA staining. Exposure to H_2_O_2_ caused a significant decrease in the percentage of intact acrosomes (43.50% [40.50–46.5%]) compared to the CTR (73.00% [67.25–76.5%], *p* < 0.01), CAF (75.50% [71.00–79.25%], *p* < 0.01), and H_2_O_2_ + CAF (65.00% [61.00–68.25%], *p* < 0.05) (Figure 5). At the same time, H_2_O_2_ treatment led to a significant increase in the percentage of altered acrosomes (32.50% [28.50–37.25%]) compared to that of CTR (20.50% [17.75–23.25%], *p* < 0.01) and CAF (18.50% [17.25–23.50%], *p* < 0.01) (Figure 5). The percentage of reacted acrosomes was significantly increased in the H_2_O_2_-treated spermatozoa (23.50% [20.75–24.75%]) compared to all other samples (CTR: 6.50% [4.25–8.75%]; CAF: 5.00% [3.50–5.75%]; H_2_O_2_ + CAF: 5.50% [2.50–7.75%], *p* < 0.01), while in the H_2_O_2_ + CAF treatment, the values were comparable to those observed in the CTR and CAF groups (Figure 5).

**Figure 6:**
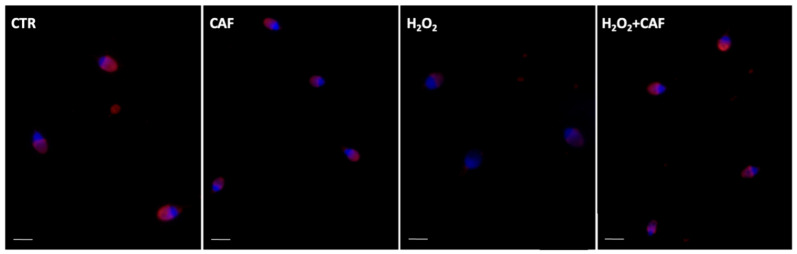
UV micrographs of human spermatozoa treated with *Pisum sativum* agglutinin (PSA) conjugated with TRITC after incubation for two hours at 37 °C with 100 μM caffeic acid (CAF), 2 mM H_2_O_2_, and combined treatment (H_2_O_2_ + CAF). CTR indicates untreated aliquot. A minimum of 300 spermatozoa were scored per sample. The acrosomes were mostly intact in the CTR and CAF aliquots; they were altered and absent in spermatozoa treated with H_2_O_2_. In the sample treated with H_2_O_2_ + CAF, the acrosomes were present, sometimes altered. Bar (CTR, H_2_O_2_): 4 µm. Bar (CAF, H_2_O_2_ + CAF): 6 µm.

In Figure 6, the PSA staining of the spermatozoa from the four treatments is represented.

## 4. Discussion

This study investigated the protective and antioxidant effects of CAF on human spermatozoa under both physiological conditions and OS induced in vitro by H_2_O_2_. Overall, the findings demonstrate that CAF exerts beneficial effects on key sperm quality parameters—namely progressive motility, and DNA and acrosomal integrity—and modulates the F_2_-IsoP concentration and the expression of genes involved in the antioxidant response. CAF, a natural occurring hydroxycinnamic acid, is contained in coffee (also decaffeinated varieties), red wine, and other plant products such as chokeberries, apples, and plums, as well as herbs like sage and oregano—members of the mint family [5]. CAF has been extensively used as a potent antioxidant in animal semen preservation [30], improving post-thaw motility and membrane integrity in boar semen [16] and enhancing semen quality in buffalo bull following freezing–thawing procedures [17]. In addition, in vivo administration of CAF in mice improved spermatogenesis by reducing OS and enhancing endogenous antioxidant defenses, thereby mitigating the adverse effects of aging—an issue of growing relevance given its documented association with declining sperm quality [15].

Despite the great number of studies for CAF’s effects on animal male fertility, only a few studies have reported its effects in human semen. Human spermatozoa represent an ideal in vitro model for evaluating antioxidant efficacy due to their availability; their limited transcriptional activity, which makes them highly sensitive to the external environment; and the ease of the assessment of functional parameters, such as motility, DNA integrity, and acrosomal status.

Various antioxidants, especially phenolic compounds found in fruits and vegetables, have been tested using this model [18,27,31]. Our research group demonstrated that 100 µM chlorogenic acid, an ester of CAF and quinic acid, exerted significant in vitro antioxidant and protective effects on swim-up selected human spermatozoa when administered either as a free molecule [22] or encapsulated in liposomes [32], as well as on whole semen samples [27].

The use of antioxidants during semen handling is particularly relevant given that procedures commonly employed during assisted reproductive technologies (ARTs) can promote reactive oxygen species overproduction and OS, if not adequately balanced by antioxidant systems [20,33]. To address this issue, several strategies have been explored. Despite considerable interest in oral antioxidant supplementation as a therapeutic approach, robust clinical evidence confirming its efficacy is still controversial [34,35]. In contrast, the direct supplementation of semen with antioxidants in vitro offers a more targeted approach to limiting reactive oxygen species-induced damage during laboratory handling.

To the best of our knowledge, the only paper on the in vitro effect of CAF (in the form of CAF phenethyl ester) on human spermatozoa reported a protective effect of this compound against DNA oxidative damage in human spermatozoa [36].

In this study, human semen samples with normal parameters were supplemented in vitro with CAF to evaluate its antioxidant and protective effects in a physiological environment—that is, the seminal plasma. Many previous studies have focused on spermatozoa isolated by swim-up or similar selection techniques that, while providing a homogeneous sperm population, simultaneously remove the seminal plasma, which contains most antioxidant defenses. By using whole semen under standardized conditions and selecting normozoospermic donors, our approach aimed to preserve an environment as close to the physiological one as possible.

A preliminary dose–response analysis using a CAF concentration range from 50 µM to 500 µM was performed based on previous literature [16,30,36]. The concentration of 100 µM was selected for other experiments, as it elicited a moderate enhancement in progressive motility without inducing detectable DNA damage. Progressive motility is essential for natural fertilization and effective sperm selection in ARTs [37]. Likewise, DNA integrity is critical for embryo development, and its impairment is associated with miscarriage and abnormal fertilization outcomes [38]. Other studies dealing with semen and spermatozoa have identified the 100 µM CAF concentration as optimal. For instance, Namula and colleagues [16] supplemented the extender used during boar semen freezing with CAF at different concentrations and found that CAF at 100 µM yielded the most pronounced improvements in post-thaw sperm quality in terms of motility, vitality, and membrane integrity. Similar results were obtained supplementing buffalo semen extenders with CAF at 100 µM [17].

The concentration of polyphenols to be used in this kind of experiment is a nuanced topic since many of these compounds can exert dose-dependent toxic effects, reducing motility and viability and increasing reactive oxygen species production at high concentrations [39]. It is not a coincidence that high concentrations of CAF (1 mM) have been used to induce apoptosis in tumor cells [40]. This suggests how moderate polyphenol levels can exert antioxidant and cytoprotective effects, whereas elevated doses may shift CAF’s action toward pro-oxidant or cytotoxic activity, underscoring the compound’s dual redox nature.

In the second step of this study, we examined the influence of CAF at 100 µM on spermatozoa exposed to in vitro-induced OS. Treatment with CAF increased the percentage of progressively motile sperm while reducing non-progressively motile sperm, although its effect on non-progressive motility remained limited when CAF was combined with H_2_O_2_. This may be due to the irreversible oxidation of thiol groups on flagellar proteins such as outer dense fiber protein 1 [41], which antioxidants cannot fully repair.

To investigate the antioxidant potential of CAF in human semen, lipid peroxidation was assessed by measuring seminal F_2_-IsoPs. These molecules, formed from arachidonic acid via reactive oxygen species-mediated peroxidation, are reliable markers of oxidative damage in spermatozoa and seminal plasma [42]. Elevated seminal F_2_-IsoP levels have been consistently associated with decreased sperm motility, vitality, and normal morphology, as well as with pathological conditions such as varicocele and urogenital infections [43,44,45]. In this study, treatment of human semen with H_2_O_2_ led an increase in F_2_-IsoP levels and co-treatment with CAF significantly decreased their concentration. This decline in seminal F_2_-IsoP level following antioxidant treatment can be considered an objective biochemical marker of reduced OS and improved sperm function. The results of this study confirm the observations obtained with a similar protocol and reported in a recent paper [27] in which chlorogenic acid, an antioxidant compound derived from CAF, restored the functional competence of spermatozoa by mitigating lipid-oxidative damage.

Regarding DNA integrity, CAF displayed protective effects by reducing H_2_O_2_-induced damage. Such oxidative damage in semen and sperm has been documented since early investigations in the field [46]. Recent evidence further confirmed that H_2_O_2_ plays a negative role in mitochondrial function and motility but also induces oxidative DNA fragmentations that compromise chromatin stability and can reduce the sperm’s ability to support normal embryo development [47]. In this context, the ability of antioxidant compounds to mitigate H_2_O_2_-induced DNA damage represents a crucial protective mechanism. By scavenging reactive oxygen species and stabilizing redox homeostasis, these agents can preserve both the structural integrity of sperm chromatin and the functional parameters essential for fertilization.

Similarly, CAF preserved acrosomal integrity. It is known that H_2_O_2_ exposure increases the proportion of spermatozoa with reacted or damaged acrosomes in both humans [46,47,48,49,50] and rats [51]. Co-treatment with CAF protected the acrosome, and this protection is particularly important, as the acrosome reaction must occur only near the oocyte, rather than prematurely within the semen. Similarly, the polyphenol quercetin has also been shown to exert protective effects on sperm structure, including the acrosome, under oxidative or inflammatory stress [52]. Together, these findings underscore that several polyphenols can contribute to defending the acrosome during sperm exposure to physiological or experimental stressors.

The mechanism of action of CAF includes the scavenging properties arising from its catechol structure, which acts as a potent antioxidant by donating hydrogen atoms to neutralize free radicals [53].

In addition, we explored the cell-signaling pathway involving Nrf2, because recently, some of our group demonstrated the effect of a similar antioxidant, chlorogenic acid, on Nrf2 gene expression [27]. Nrf2 levels positively correlate with key sperm quality parameters, including concentration, motility, and vitality. Moreover, Cheng et al. [54] reported that, in cases of asthenozoospermia, low Nrf2 mRNA expression was associated with reduced seminal SOD activity and lower mRNA levels of CAT and SOD2 (although these decreases were not statistically significant).

The critical role of Nrf2 in protecting spermatogenesis from oxidative damage was also confirmed in Nrf2-knockout mice, generated by Nakamura and colleagues in 2010 [55]. In these models, deletion of Nrf2 resulted in significantly reduced sperm concentrations in both the testis and epididymis, along with a decrease in epididymal sperm motility, compared to wild-type mice.

In this study, we observed that CAF modulated the Keap1–Nrf2–HO-1 signaling pathway in vitro. Although spermatozoa have minimal transcriptional activity due to their highly condensed chromatin, increasing evidence suggests that a residual gene expression can occur in response to environmental stimuli [56]. Several studies demonstrated a modulation of sperm transcription in vitro using antioxidants, such as melatonin [57], curcumin [58], and chlorogenic acid [27]. The modulation of the Keap1-Nrf2 pathway suggests the activation of the ARE pathway, which leads to the transcription of cytoprotective genes such as HO-1 [59,60]. The observed upregulation of Nrf2 and HO-1 suggests the activation of endogenous antioxidant defenses. Despite the trend of Keap1 expression being similar to those of the other analyzed genes, the lack of all of those statistically significant changes in Keap1 is not unexpected, as Keap1’s activity is primarily regulated through conformational and post-translational modifications rather than through alterations in its expression levels. Under OS, Keap1 undergoes conformational changes via modification of key cysteine residues (e.g., Cys151, Cys273, Cys288), which functionally inactivate its ability to ubiquitinate Nrf2, stabilizing it and promoting its nuclear translocation [61,62,63]. As a result, newly synthesized Nrf2 escapes Keap1-mediated degradation, accumulates in the nucleus, and upregulates downstream antioxidant genes such as HO-1 [64].

In addition to the Nrf2 pathway, CAF acts as a regulatory compound modulating numerous biochemical pathways such as NF-κB, STAT3, and ERK1/2; the FOXO1/FIS pathway; tumor necrosis factor-α; interleukin-6; and cyclooxy-genase-2, thereby reducing inflammatory responses [6,8,11,13], which would also be interesting to investigate in spermatozoa.

We acknowledge that the study has several limitations, which include the relatively limited sample size. Increasing the number of cases would strengthen the statistical power and robustness of the results. Moreover, the assessment of DNA integrity could be further developed by applying more sensitive and advanced analytical methods. The investigation of additional molecular pathways potentially involved in the observed effects would provide a more comprehensive understanding of the underlying mechanisms. Finally, the effect of CAF on sperm capacitation could be an interesting issue to explore.

Based on the data currently available to us, treatment with CAF does not appear to influence the capacitation process; however, it would also be interesting to explore whether CAF could have a de-capacitating effect.

Further research is needed to confirm the potential of CAF as a supplement during in vitro semen handling and to evaluate whether in vitro improvements in sperm parameters translate into enhanced fertilization and pregnancy outcomes. In addition, studies should also include samples beyond normozoospermic donors, as these may not fully reflect the responses of the spermatozoa of infertile patients or individuals with altered semen parameters. Finally, due to its potent antioxidant properties, CAF should be tested in semen cryopreservation protocols.

In conclusion, CAF emerges as a potent in vitro antioxidant capable of preserving key functional and structural characteristics of human spermatozoa exposed to OS in vitro. Its ability to modulate the Keap1–Nrf2–HO-1 pathway provides support for its protective role. The supplementation of CAF into semen-processing protocols may represent a promising strategy for enhancing sperm quality in research and clinical settings.

## Data Availability

The data presented in this study are available on request from the corresponding author. The data are not publicly available due to the privacy of the patients.

## Abbreviations

The following abbreviations are used in this manuscript:

OS	Oxidative Stress
CAF	Caffeic Acid
F_2_-IsoPs	F_2_-IsoProstanes
PBS	Phosphate-Buffered Saline
PSA	*Pisum sativum* Agglutinin
ARTs	Assisted Reproductive Technologies
